# The Structure, Biosynthesis, and Function of β-1,6-Glucan in the Fungal Cell Wall

**DOI:** 10.3390/biom16091233

**Published:** 2026-08-26

**Authors:** Yanxin Wang, Zhenhao Zhao, Tongyu Li, Guoqi Liu, Jiale Wang, Zhoukun Li

**Affiliations:** 1 College of Agriculture and Biology, Liaocheng University, Liaocheng 252000, China; 2 Key Laboratory of Agricultural Environmental Microbiology, Ministry of Agriculture and Rural Affairs, College of Life Sciences of Nanjing Agricultural University, Nanjing 210095, China

**Keywords:** β-1,6-glucan, fungal cell wall, structure, biosynthesis, function, cell wall biology

## Abstract

β-1,6-Glucan is a functionally crucial polysaccharide of the fungal cell wall, although typically less abundant than β-1,3-glucan and chitin, its content, chain length, and branching vary considerably among species. Structurally, it serves as a covalent cross-linker tethering the external mannoprotein layer to the internal β-1,3-glucan-chitin network, contributing to cell wall integrity and plasticity. Biosynthetically, unlike chitin and β-1,3-glucan, which are synthesized by the plasma membrane-associated synthases, β-1,6-glucan biosynthesis depends on a multi-protein cooperative network spanning the endoplasmic reticulum (ER), Golgi, and cell surface, whose core catalytic machinery remains incompletely defined. Genetic and in vitro reconstitution studies have begun to delineate the contributions of ER-resident proteins (Kre5, Big1, Cwh41/Gls1, Rot2/Gls2, and Cne1), Golgi-localized Kre6/Skn1 family members, and cell-surface components (Kre9, Knh1, Kre1, and Kre11). Functionally, its biological roles are established by two complementary lines of evidence, namely enzymatic digestion by endogenous or exogenous β-1,6-glucanases and inactivation of the biosynthetic machinery. Collectively, these studies show that β-1,6-glucan is essential for cell wall architecture, GPI-anchored protein localization, fungal growth, morphogenesis, and virulence, and acts as a potent immunomodulatory molecule at the fungus–host interface. Elucidating its structure, biosynthesis, and function will advance fungal cell wall biology.

## 1. Introduction

**Figure 1:**
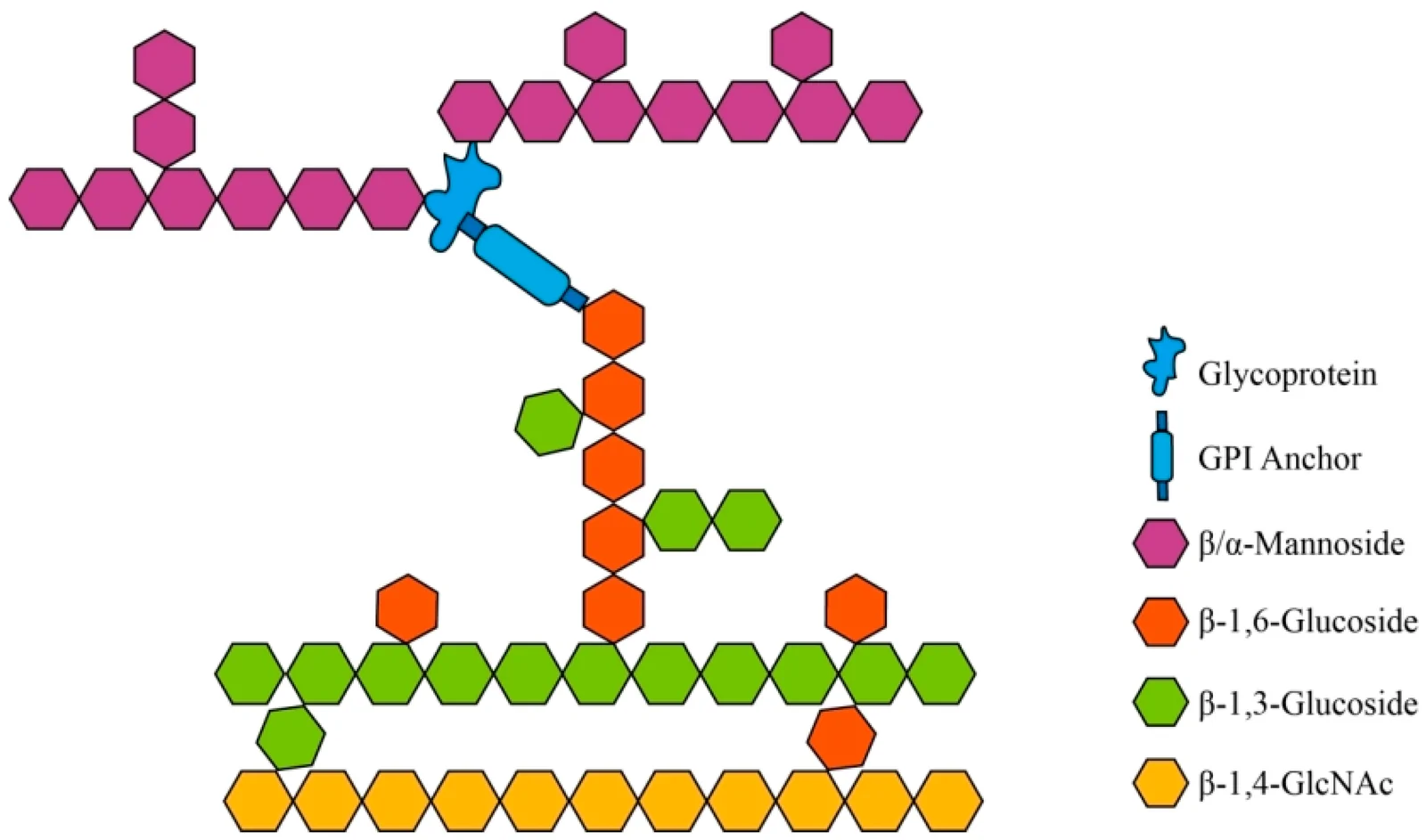
The significance of β-1,6-glucan in the fungal cell wall architecture. This polymer functions as a cross-linker, tethering mannoproteins to the cell wall core structure made of chitin and β-1,3-glucan.

The fungal cell wall is a dynamic and structurally complex organelle essential for cell morphology, viability, and pathogenicity [1,2]. It serves both as a physical barrier against external environmental stress and as a critical interface that activates host immune responses during infection [3,4]. Its conserved components represent the principal source of pathogen-associated molecular patterns (PAMPs) that trigger host immune recognition [5,6,7,8]. Therefore, comprehensively elucidating the molecular structure and assembly of the fungal cell wall is of fundamental importance, as it not only deepens our mechanistic understanding of fungal biology, but also provides a solid theoretical foundation that may ultimately support the identification and rational design of antifungal targets. Fungal cell wall typically exhibits a “rigid interior, flexible exterior” bilayer structure [9,10], despite species-specific variations in their molecular composition. The outer layer is highly plastic and comprises multiple components, including α-1,3-glucan, mannoproteins, galactomannan, and hydrophobins [11,12,13], which are primarily involved in environmental interactions and immune evasion. The internal skeletal layer is evolutionarily conserved and predominantly consists of chitin (1–20% of cell wall dry weight) and β-1,3-glucan (50–60% of dry weight) [14,15,16], which collectively provide mechanical strength and morphological stability. Additionally, the inner layer also harbors β-1,6-glucan, a polysaccharide characterized by a linear chain of glucose units connected via β-1,6-glycosidic linkages, along with a few side chains formed by β-1,3-glycosidic connections. This molecule serves as a covalent cross-linker, connecting external mannoproteins to the internal chitin-β-1,3-glucan network [17,18] (Figure 1), thereby forming a robust scaffold essential for maintaining the structural integrity of the fungal cell wall.

The biosynthesis of fungal cell wall is a complicated, multi-step process involving the coordinated action of cytoplasmic precursor synthesis, polymerization at the plasma membrane, and assembly and modification in the extracellular space. For instance, chitin synthesis is catalyzed by plasma membrane-localized chitin synthases (CHSs), which employ UDP-N-acetylglucosamine (UDP-GlcNAc) as a substrate and systematically elongate the polysaccharide chain via β-1,4-glycosidic bonds [19,20,21]. β-1,3-Glucan is synthesized by the membrane-associated β-1,3-glucan synthase catalytic subunit Fks (Fks1/Fks2), with the Rho-family small GTPase Rho1 as a regulatory subunit and UDP-glucose as the sugar donor, to form the predominant polysaccharide framework of the cell wall [22,23,24]. α-1,3-Glucan is synthesized by the plasma membrane-associated α-1,3-glucan synthase (AGS) [25,26,27]. In contrast, mannoproteins synthesis involves endoplasmic reticulum (ER)-mediated protein synthesis and Golgi-dependent glycosylation, followed by integration into the cell wall via glycosylphosphatidylinositol (GPI) anchors [28,29,30]. Many core components of the fungal cell wall lack analogous structures in plant or animal hosts, and their distinctive synthetic pathways are therefore of fundamental biological interest; a detailed characterization of these pathways may provide important theoretical insights that could ultimately inform the identification of selective antifungal targets [31,32]. However, currently available drugs that target cell wall synthesis have notable limitations. For example, echinocandins such as caspofungin non-competitively inhibit β-1,3-glucan synthase Fks, compromising cell wall integrity, yet their prolonged and extensive use has resulted in increasing drug resistance [33,34,35]. Nikkomycin Z is an efficient inhibitor of chitin synthases, causing cell wall abnormalities and hyphal malformation, but its narrow antifungal spectrum constrains its clinical utility [36,37,38]. These limitations underscore the need for a more comprehensive understanding of fungal cell wall biogenesis, which is essential for identifying novel, conserved, and broad-spectrum targets and for laying the theoretical foundation of sustainable antifungal strategies.

Among the major cell wall components, β-1,6-glucan remains one of the least understood despite its functional importance. Unlike the well-characterized core components chitin and β-1,3-glucan, β-1,6-glucan is present at a relatively low level in most fungal cell walls, although its content varies considerably among species [14,39]. Nevertheless, it is essential as a covalent cross-linker bridging the inner and outer wall layers [39,40,41,42], thereby contributing to both mechanical resilience and functional integrity. However, the molecular mechanisms underlying its synthesis remain largely unresolved. Current evidence suggests that β-1,6-glucan production requires the coordinated involvement of multiple subcellular compartments, including the ER, Golgi, and plasma membrane [32,33,34], yet the precise molecular details are unclear. This knowledge gap limits our understanding of fungal cell wall assembly and biogenesis. Given that the cell wall is a crucial interface for host–pathogen interactions [43,44,45], a thorough elucidation of β-1,6-glucan biology is of broad significance. In this review, we focus on the structure, biosynthesis, and function of β-1,6-glucan in the fungal cell wall, systematically summarizing current knowledge on this polymer. We anticipate that a deeper understanding of the biological roles and regulatory network of β-1,6-glucan will not only advance our knowledge of cell wall biogenesis and assembly principles but also provide important theoretical and genetic resources that may inform the rational development of novel antifungal strategies.

## 2. Structural Diversity of β-1,6-Glucan in the Fungal Cell Wall

β-1,6-glucan is typically a minor yet functionally crucial component of the fungal cell wall. Its content and arrangement have been investigated in *Saccharomyces cerevisiae* and several human-pathogenic fungi (Table 1). In *S. cerevisiae*, it is a highly branched polymer of about 350 glucose residues, with an average molecular weight of approximately 57 kDa, constituting 15% of the cell wall dry weight [46]. In *Malassezia restricta*, this polymer constitutes up to 70% of total cell wall polysaccharides, with an average molecular weight of approximately 80 kDa, and is found almost exclusively in the alkali-insoluble fraction [47]. In *Candida albicans*, it accounts for around 23% of the cell wall dry weight, its chain length and branching degree are dynamically regulated by environmental conditions. Under hyphal morphology, the average chain length is 43 kDa, lower than in yeast-form cells, but increases to 70 kDa upon treatment with the antifungal drug caspofungin [48]. In contrast, β-1,6-glucan exhibits a morphotype-specific distribution in *Aspergillus fumigatus*, being found solely in the conidial cell wall in low levels, while remaining entirely absent in the mycelial cell wall [49]. This strict conidium-restricted localization not only implies a potential role of this polymer in fungal developmental transitions, but also suggests the plasticity of cell wall architecture across the fungal life cycle and the species-specific regulation of β-1,6-glucan synthesis.

## 3. Biosynthesis of β-1,6-Glucan: Associated Proteins and Unresolved Enigmas

### 3.1. Overview and Associated Proteins

Before discussing the biological functions of β-1,6-glucan, it is instructive to summarize how this polymer is produced, since the functional consequences described below are best understood in light of the biosynthetic machinery. To date, the primary catalytic proteins responsible for β-1,6-glucan polymerization, as well as their regulatory mechanisms have not been definitively identified. The synthesis of β-1,6-glucan is recognized as a complex, multi-stage process that depends on the secretory pathway, involving a cascade of functionally diverse proteins localized in the ER, Golgi and cell surface. The majority of these proteins have been functionally characterized in *S. cerevisiae*, as well as in a few plant pathogenic fungi like *Colletotrichum graminicola*, and in several clinically relevant human pathogens like *Cryptococcus neoformans*, *C. albicans*, *Candida auris*, *Candida glabrata*, and *A. fumigatus*. The effects of these proteins on fungal cell wall composition, development and virulence have been thoroughly delineated (Table 2), based on subcellular localization and genetic analyses performed to date.

#### 3.1.1. ER-Associated Proteins

ER is the initiating site for β-1,6-glucan synthesis. Several ER-resident proteins, including Kre5, Big1, Cwh41/Gls1, Rot2/Gls2 and Cne1, are involved primarily in substrate preparation and quality control. Rather than directly catalyzing β-1,6-glucan polymerization, these proteins function in the processing and maturation of N-linked glycans, the recognition and refolding of misfolded glycoproteins, and the proper assembly or localization of key biosynthetic enzymes. Together, they ensure that correct precursors and functional complexes are available for subsequent cell surface glucan synthesis.

**Table 2:** Proteins implicated in β-1,6-glucan synthesis and the phenotypic effects of their deletion on fungal growth and development.

Location	Protein	Fungus Species	Special Name	No. of Homologs	Cell Wall Composition Changes	Growth and Development	References
ER	Kre5	*S. cerevisiae*	ScKre5	1	β-1,6-Glucan absent (0); β-1,3-glucan increased; mannoprotein severely reduced (0) with defective α-agglutinin anchoring	Severely slowed growth rate; occurrence of large, multi-budded cells; incomplete cell separation; intracellular accumulation of ER membranous and vacuolar structures	[50]
		*C. albicans*	CaKre5	1	β-1,6-Glucan decreased (20); chitin increased (200); β-1,3-glucan increased (150); mannoprotein decreased (32)	Markedly impaired growth or mortality; cellular aggregation; expanded vacuoles; complete inability to form hyphae; loss of pathogenicity	[51]
		*C. glabrata*	CglKre5	1	β-1,6-Glucan decreased (50); chitin increased (500); β-1,3-glucan unchanged	Decreased growth rate; cellular aggregation; irregular cell morphology; impaired cell wall integrity; increased susceptibility to environmental stresses	[52]
		*C. neoformans*	CnKre5	1	β-1,6-Glucan absent (0); chitin unchanged; chitosan redistribution (reduced at septum, accumulated laterally); mannoprotein decreased; GPI-AP Plb1 wall retention reduced; enlarged and diffuse EPS capsule	Decreased growth rate; increased cell size; cell aggregation; large intracellular vacuole/vesicle structures; aberrant multiple budding; incomplete daughter cell separation (cell chains); loss of virulence	[53]
		*C. graminicola*	CgKre5	1	β-1,6-Glucan and β-1,3-glucan decreased	Severely compromised growth; increased cellular size; swollen vegetative hyphae with intrahyphal hyphae and recurrent rupture releasing lipid vesicles; aberrant fragile conidial morphology; spontaneous appressorium lysis; enhanced cell wall elasticity; diminished turgor pressure; impaired penetration; loss of virulence	[54]
	Big1	*S. cerevisiae*	ScBig1	1	β-1,6-Glucan decreased (5)	Severely compromised growth; cell aggregation; increased susceptibility to SDS and hygromycin B	[55]
		*C. albicans*	CaBig1	1	β-1,6-Glucan decreased (50); chitin increased (120); β-1,3-glucan unchanged	Impaired hyphal development; diminished host adherence; severely attenuated pathogenicity	[56]
	Cwh41/Gls1	*S. cerevisiae*	ScCwh41/Gls1	1	β-1,6-Glucan decreased (50); chitin, β-1,3-glucan and mannoprotein unchanged	No obvious growth defect	[57]
	Rot2/Gls2	*S. cerevisiae*	ScRot2/Gls2	1	β-1,6-Glucan decreased (55); chitin, β-1,3-glucan and mannoprotein unchanged	No obvious growth defect	[58]
	Cne1	*S. cerevisiae*	ScCne1	1	β-1,6-Glucan decreased (70); chitin, β-1,3-glucan and mannoprotein unchanged	No obvious growth defect	[46]
		*C. glabrata*	CglCne1	1	β-1,6-Glucan decreased (70); chitin increased (260); β-1,3-glucan unchanged	Exacerbated growth defect; cell agglutination	[59]
Golgi	Kre6/Skn1	*S. cerevisiae*	ScKre6	2	β-1,6-Glucan reduced (50); β-1,3-glucan increased; mannoprotein severely reduced (0) with defective α-agglutinin anchoring	Substantially retarded growth; cellular aggregation; expanded cellular morphology	[60]
			ScSkn1		β-1,6-Glucan, chitin, β-1,3-glucan and mannoprotein remain unchanged	No obvious growth defect	
		*C. albicans*	CaKre6, CaKre62, CaSkn1, CaSkn2	4	β-1,6-Glucan absent (0); chitin increased (380); mannoprotein absent (0); defective localization of GPI-anchored proteins;	A longer lag phase; enlarged cell phenotype; stunted growth; filamentation defects; severe cell separation defects; irregular cell wall ultrastructure; increased vulnerability to cell wall toxins; compromised biofilm formation; diminished pathogenicity	[48,61,62]
		*C. neoformans*	CnKre6 and CnSkn1	6	β-1,6-Glucan absent (0); chitin unchanged; chitosan relocation; GPI-AP Plb2 wall retention reduced; enlarged and diffuse EPS capsule	Reduced growth rate; increased cell size; cell aggregation; large intracellular vacuoles/vesicles; aberrant multiple budding; incomplete daughter cell separation (cell chains); diminished pathogenicity	[53]
		*C. graminicola*	CgKre6	1	β-1,6-Glucan and β-1,3-glucan decreased	Pronounced growth impairment; increased cell volume; distended vegetative hyphae with intrahyphal hyphae and frequent rupture; irregular fragile conidial structure; spontaneous lysis of appressoria; augmented cell wall elasticity; diminished turgor pressure; impaired penetration; loss of pathogenicity	[54]
		*A. fumigatus*	AfKre6	1	β-1,6-Glucan absent (0); chitin remains unchanged; β-1,3-glucan increased (104); β-1,3/1,4-glucan increased (102)	Unaltered growth rate; increased susceptibility to cell wall stresses (Congo Red and Calcofluor White)	[49]
		*C. auris*	CaKre6a	2	β-1,6-Glucan reduced (40–50); β-1,3-glucan increased; chitin and mannoprotein remain unchanged; *KRE6b* downregulated	Reduced echinocandin susceptibility; accumulated chitin and retained rigid β-1,3-glucan under stress; its deletion downregulated *KRE6b*.	[63]
			CaKre6b			Weaker cell walls with lower chitin and overproduced β-1,6-glucan under stress; has no effect on *KRE6a* expression	
Cell surface	Kre11	*S. cerevisiae*	ScKre11	1	β-1,6-Glucan reduced (50); chitin, β-1,3-glucan and mannoprotein remain unchanged; defective cell wall anchorage of α-agglutinin	No obvious growth defect	[64]
	Kre1	*S. cerevisiae*	ScKre1	1	β-1,6-Glucan reduced (60); chitin, β-1,3-glucan and mannoprotein remain unchanged; defective cell wall anchorage of α-agglutinin	Irregular cell surface morphology	[65]
	Kre9	*S. cerevisiae*	ScKre9	1	β-1,6-Glucan reduced (20); chitin, β-1,3-glucan and mannoprotein remain unchanged; defective cell wall anchorage of α-agglutinin	Greatly suppressed growth; aberrant multiple-budded morphology; deficiency in polarized cell wall development	[64]
	Knh1	*S. cerevisiae*	ScKnh1	1	β-1,6-Glucan and other cell wall components remain unchanged	No obvious growth defect	[66]

*S. cerevisiae*, *Saccharomyces cerevisiae; C. albicans*, *Candida albicans; C. glabrata*, *Candida glabrata; C. neoformans*, *Cryptococcus neoformans; C. graminicola*, *Colletotrichum graminicola*; A. fumigatus, Aspergillus fumigatus. C. auris, Candida auris. No. of Homologs, number of homologous proteins. EPS, Exopolysaccharides; GPI-AP, GPI-anchored proteins. Values in Cell wall composition changes columns indicate the percentage of the component in mutant relative to wild-type (wild-type = 100).

Kre5 is a critical ER protein homologous to mammalian UDP-glucose:glycoprotein glucosyltransferase (UGGT) [67,68]. Based on its structural features, it is proposed to function as a glucosyltransferase that mediates N-glycan reglucosylation in the endoplasmic reticulum, a process that adds glucose residues to N-linked glycans of proteins to facilitate glycoprotein folding quality control [69,70]. This modification is considered an essential prerequisite for subsequent elongation and synthesis of β-1,6-glucan chains. Deletion of *KRE5* results in a complete loss of β-1,6-glucan in the cell wall of *S. cerevisiae*, accompanied by severe growth defects and potential lethality [50]. Moreover, the essential function of Kre5 has been corroborated in other human pathogenic fungi, including *C. albicans*, *C. neoformans* and *C. glabrata*, where its deletion leads to a substantial decrease or complete loss of β-1,6-glucan content [51,52,53]. Functional conservation of Kre5 has been observed in the plant pathogenic fungus *C. graminicola*, RNA interference-mediated downregulation of its expression results in a profound reduction in β-1,6-glucan content and induces multiple developmental and pathogenicity defects [54]. Collectively, these data indicate that the Kre5 protein is evolutionarily conserved in both human and plant pathogenic fungi.

Big1p is an integral membrane protein localized in the ER. In *S. cerevisiae*, deletion of *BIG1* results in an approximately 95% reduction in β-1,6-glucan, and the deletion strain exhibited a phenotype similar to that of the Δ*kre5* mutant [55]. In *C. albicans*, *CaBIG1* deletion leads to reduced β-1,6-glucan levels and hyphal development defects [56]. Moreover, the concurrent deletion of Big1 and Kre5 in *S. cerevisiae* exacerbates the phenotypic abnormalities observed in single deletion strains of either gene [55], indicating that the two proteins function through partially independent pathways to support normal β-1,6-glucan synthesis, but the detailed role of Big1 in β-1,6-glucan metabolism remains to be elucidated.

Cwh41p/Gls1 and Rot2p/Gls2 are glucosidase I and glucosidase II enzymes, respectively, both localized in the ER, where they remove glucose residues from N-linked glycan chains [46,71]. These two proteins function together with the UGGT homolog Kre5 in the N-glycan deglucosylation and reglucosylation cycle within the ER. The elimination of Cwh41 and Rot2 reduces β-1,6-glucan level by 50% in *S. cerevisiae* cell wall, but does not induce impairments in growth or cell wall protein anchoring [57,58]. Therefore, Cwh41, Rot2, and Kre5 are all hypothesized to influence β-1,6-glucan synthesis indirectly, as the untrimmed or aberrantly processed N-linked glucose residues may sterically hinder the binding of β-1,6-glucan to protein N-glycans, or may disrupt the proper folding of key proteins required for β-1,6-glucan assembly. Cne1, a calnexin homolog, participates in the quality control of protein folding within the ER. Elimination of *CNE1* reduces β-1,6-glucan by 30% in the cell walls of both *S. cerevisiae* and *C. glabrata* [59,72]. While this deletion has no effect on growth or cell wall protein anchoring in *S. cerevisiae* [72], it leads to growth defects and cell aggregation in *C. glabrata* [59].

#### 3.1.2. Golgi-Associated Proteins

Kre6 and Skn1 are homologous type II transmembrane proteins localized in the Golgi apparatus and are functionally redundant. The number of Kre6 homologs, however, varies considerably across fungal species, such as two in *S. cerevisiae* (ScKre6 and ScSkn1) [60], four in *C. albicans* (CaKre6, CaSkn1, CaKre62 and CaSkn2) [48,61,62], six in *C. neoformans* (CnKre6, CnSkn1, CnKre61, CnKre62, CnKre63 and CaKre64) [53], and only one in *C. graminicola* (CgKre6) [54] and *A. fumigatus* (AfKre6) [49]. This remarkable diversity in Kre6 homologs across the fungal kingdom suggests lineage-specific functional expansion and specialization of β-1,6-glucan synthesis. In *S. cerevisiae*, single deletion of *KRE6* leads to an approximately 50% reduction in β-1,6-glucan, accompanied by impaired growth and morphological defects, whereas single deletion of *SKN1* causes no detectable phenotypic defects [60]. However, the simultaneous deletion of both proteins severely compromises β-1,6-glucan production and exacerbates the phenotypic defects of the deletion strain. Kre6 and Skn1 exhibit sequence homology with bacterial β-1,3-glucanases [46], suggesting a potential role in the processing or elongation of glucan chains during β-1,6-glucan synthesis. In *C. albicans*, single deletions of Skn1, Kre62, or Skn2 cause no pronounced changes in cell wall composition, whereas Kre6 and Kre6/Skn1 double mutants both exhibit a 46.8% reduction in β-1,6-glucan, and these two mutants produce β-1,6-glucans that are shorter and more highly branched, suggesting that these proteins are involved primarily in glucan chain elongation rather than branching. Importantly, the quadruple deletion of all four homologs (Kre6/Kre62/Skn1/Skn2) caused complete absence of β-1,6-glucan [48]. Additionally, the concurrent deletion of *KRE6* and *SKN1* completely abolishes β-1,6-glucan synthesis in *C. neoformans* [53]. Besides that, two contiguous β-1,6-glucan synthesis-related genes *KRE6a* and *KRE6b* were identified in *C. auris*, exhibiting 57% homology to *C. albicans KRE6*. Their essentiality for *C. auris* viability was evidenced by the failure of repeated double-knockout attempts, in which both genes persisted despite successful cassette integration, and single deletion of Kre6a or Kre6b resulted in a 40–50% reduction in β-1,6-glucan content [63,73]. Notably, a strong auto-regulatory loop of Kre6a over Kre6b was observed, suggesting a hierarchical control mechanism between these two synthesis-related genes [63,73]. These findings indicate that Kre6 and Skn1 are essential mediators of β-1,6-glucan synthesis.

#### 3.1.3. Cell Surface and Cytoplasmic Proteins

Kre9p and Knh1p are homologous cell surface proteins. Deletion of *KRE9* in *S. cerevisiae* results in diminished β-1,6-glucan levels and budding defects [64,66]. Kre9 and Knh1 may function as regulatory elements in β-1,6-glucan polymer synthesis, contributing to the attachment of the synthesized polymer to the cell wall [46]. Kre1, a putative GPI-anchored protein, is localized at the cell surface [74]. Its deletion also leads to a moderate decrease in β-1,6-glucan [65]. Kre11 is a cytoplasmic protein belonging to the TRAPP complex, which is involved in the docking and fusion of ER-derived vesicles with the Golgi apparatus [75]. This protein is proposed to indirectly influence the transport of β-1,6-glucan biosynthetic components through the secretory pathway by modulating vesicle trafficking [76]. Additionally, genomic studies indicate that *KRE9*, *KNH1*, *KRE1*, and *KRE11* may be exclusive to ascomycetes, including *S. cerevisiae* and *Candida* species, while being absent in basidiomycetes like *C. neoformans* [53], demonstrating that the synthesis processes of β-1,6-glucan vary among different fungal taxa.

### 3.2. In Vitro Reconstitution Studies

Although the genetic and localization analyses described above have identified a panel of proteins involved in β-1,6-glucan biosynthesis, the core catalytic synthases and their precise biochemical functions have remained elusive. Two landmark in vitro reconstitution studies have helped bridge this gap by providing fundamental biochemical insights into the polymerization process. Vink et al. (2004) developed an in vitro assay using crude membrane preparations from *S. cerevisiae*, demonstrating that β-1,6-glucan synthase activity is time- and GTP-dependent and utilizes UDP-glucose as the direct sugar donor [77]. They further identified the small GTPase Rho1p as a positive regulator of this activity, revealing a regulatory node shared with the β-1,3-glucan synthase system [77]. Building on this work, Aimanianda et al. (2009) established a permeabilized cell-based assay that preserved cellular ultrastructural integrity, a prerequisite for β-1,6-glucan synthase activity [78]. Using this system, they confirmed that the in situ-synthesized polymer is chemically and structurally identical to native cell wall β-1,6-glucan, comprising a β-1,6-linked backbone with β-1,3-linked side chains [78]. Notably, the complete loss of activity observed in Kre5 and Kre9 deletion mutants provided direct biochemical evidence linking these gene products to β-1,6-glucan synthesis [78]. Importantly, both studies converge on the conclusion that UDP-glucose serves as the essential substrate for β-1,6-glucan polymerization, establishing a shared biosynthetic precursor with β-1,3-glucan. The strict dependence of β-1,6-glucan synthase activity on preserved cellular integrity further underscores the complexity of its biosynthetic machinery and suggests that the native enzyme complex may require specific membrane environments, protein–protein interactions, or subcellular compartmentalization that cannot be recapitulated by simple detergent-solubilized systems.

## 4. Function of β-1,6-Glucan in the Fungal Cell Wall

### 4.1. Complementary Approaches to Dissecting β-1,6-Glucan Function

Current knowledge of β-1,6-glucan function rests on two complementary lines of evidence. The first relies on enzymatic digestion, in which endogenous or exogenous β-1,6-glucanases have been used to selectively remove β-1,6-glucan, and the resulting phenotypic changes are interpreted in terms of polymer function (Table 3). However, because β-1,6-glucan covalently cross-links mannoproteins to the β-1,3-glucan-chitin network, its enzymatic removal may secondarily expose or destabilize other cell wall components, such as β-1,3-glucan, chitin, or mannoglycoconjugates. The effects observed after digestion are therefore not always attributable solely to the loss of β-1,6-glucan itself, and digestion-based evidence is best regarded as supportive rather than definitive. The second, and more direct, line of evidence derives from the inactivation or inhibition of the β-1,6-glucan synthesis-related proteins by gene deletion (Table 2), an approach that bypasses the confounding effects inherent to enzymatic digestion. Notably, the two tables cover partly different fungal species. In genetically tractable organisms such as *S. cerevisiae*, *C. albicans*, *C. neoformans*, function is primarily demonstrated by gene inactivation, whereas in species refractory to genetic manipulation, such as the basidiomycetes *Lentinula edodes* and *Coprinopsis cinerea*, function is largely inferred from digestion experiments. Together, these complementary approaches provide a robust picture of β-1,6-glucan function in cell wall architecture, fungal development, and host interaction.

### 4.2. Structural Function: Cell Wall Integrity and Architecture

#### 4.2.1. Evidence from Enzymatic Digestion

Among the digestion-based evidence discussed above, endogenous β-1,6-glucanases, enzymes produced by fungi themselves to remodel their own cell walls, play pivotal roles in fungal growth, development, and differentiation (Table 3). In basidiomycetes, β-1,6-glucan serves as a critical structural element of the fruiting body cell wall. The endo-β-1,6-glucanase LePus30A derived from the fruiting body of *L. edodes* is highly expressed during postharvest autolysis, suggesting that regulated β-1,6-glucan degradation is critical for fruiting body senescence [79,80]. Similarly, the endo-β-1,6-glucanase GH30A from *C. cinerea* selectively hydrolyzes β-1,6-glucan in the stipe cell wall, releasing glucose and gentiobiose and thereby restoring cell wall extensibility in heat-inactivated stipes [81,82]. This finding implies the existence of cross-links between β-1,6-glucan and chitin, and demonstrates that these interactions are essential for cell wall relaxation during stipe elongation [82]. In the plant pathogenic fungus *Magnaporthe oryzae*, knockout of the endogenous β-1,6-glucanase MoGlu16 significantly reduces mycelial growth rate, decreases β-1,6-glucan content by 13.7%, increases chitin content by 12.2%, enhances susceptibility to cell wall stressors, and severely impairs pathogenicity [83,84]. Likewise, deletion of the endogenous β-1,6-glucanase VfGlu1 in the mushroom pathogen *Verticillium fungicola* markedly reduced its pathogenicity towards *Agaricus bisporus* [8,85]. Collectively, these findings indicate that fungi employ endogenous β-1,6-glucanases to precisely regulate β-1,6-glucan metabolism, and this regulation is directly coupled to cell wall remodeling, hyphal extension, and developmental transitions. Interference with this endogenous β-1,6-glucan modification machinery consistently impairs fungal growth and development (Table 3), underscoring the central role of β-1,6-glucan as a dynamic regulator of fungal morphogenesis.

In addition to the endogenous regulation described above, exogenous β-1,6-glucanases that degrade β-1,6-glucan to suppress fungal growth provide further, albeit indirect, evidence of the indispensable role of this polymer in cell wall integrity (Table 3). For instance, GluM, an outer membrane β-1,6-glucanase derived from the myxobacterium *Corallococcus* sp. EGB, effectively degrades the cell wall of *M. oryzae*, causing aberrant hyphal morphology, altered chitin distribution, increased membrane permeability, and suppressed fungal growth and pathogenicity [86,87]. Similarly, FlGlu30, a β-1,6-glucanase derived from the endophytic bacterium *Flavobacterium* sp. NAU1659, damages the *M. oryzae* cell wall by specifically hydrolyzing β-1,6-glucan, resulting in cell membrane degradation, accumulation of reactive oxygen species (ROS), and complete inhibition of conidial germination and appressorium formation [88]. The fact that sole degradation of β-1,6-glucan by a single exogenous enzyme is sufficient to induce severe cellular dysfunction powerfully underscores the indispensable role of this polymer in fungal cell biology. Beyond bacterial sources, β-1,6-glucanases derived from the biocontrol fungus *Trichoderma harzianum*, specifically BGN16.3 and Neg1, effectively suppress plant pathogenic fungi through synergism with other cell wall-degrading enzymes, particularly chitinases [75,77,78,79,89,90,91]. This collaborative activity further confirms that β-1,6-glucan serves as a crucial cross-linking element within the cell wall matrix, and its elimination destabilizes the entire wall architecture, thereby compromising fungal viability. Together, these results indicate that β-1,6-glucan represents a vulnerable Achilles heel of the fungal cell wall, such that compromising its integrity leads to severe growth impairment and reduced virulence in fungi.

#### 4.2.2. Evidence from Inactivation of β-1,6-Glucan Biosynthesis

As a second and more direct line of evidence, the inactivation or inhibition of β-1,6-glucan synthesis-related proteins, such as Kre5 and Big1 in *S. cerevisiae* [50,55], Kre6/Skn1/Kre62/Skn2 in *C. albicans* [48], and Kre5 and Kre6/Skn1 in *C. neoformans* [53], leads to alterations in cell wall composition, impaired GPI protein anchoring, and ultrastructural anomalies, as detailed in Table 2. These defects provide critical insights into the specific function of β-1,6-glucan in the cell wall. To prevent deterioration of the skeletal polysaccharide network, fungi activate the cell wall integrity (CWI) pathway, triggering compensatory remodeling. This response, characterized by elevated chitin and β-1,3-glucan, is conserved in both *S. cerevisiae* and *C. albicans*. In *S. cerevisiae*, mutants lacking Kre5, Big1, or Kre6 exhibit this characteristic remodeling [50,55,60]. In *C. albicans*, Kre6 deletion reduced β-1,6-glucan by nearly half and mannan by over forty percent, while chitin increased nearly fourfold and β-1,3-glucan approximately twofold, with remaining β-1,6-glucan chains becoming shorter and more branched; complete loss of β-1,6-glucan in the quadruple Kre6-family mutant raised chitin to nine point five percent and β-1,3-glucan branching to forty-eight percent [48]; similarly, Kre5 and Rot2 deletions caused β-1,6-glucan reduction exceeding eighty percent, with compensatory chitin and β-1,3-glucan increases [48]. Furthermore, inhibition of Kre6/Skn1 activity activates the Ca^2+^-calcineurin pathway in *C. albicans*, which acts upstream of the Pkc1-Mkc1 MAPK pathway and leads to increased chitin content through the post-transcriptional regulation of the chitin synthase Chs3, as reported by Han et al. [61,62]. Notably, concurrent deletions of Chs3 and Kre6/Skn1 in *C. albicans* result in cell death [61,62], indicating that this compensatory pathway is essential for survival when β-1,6-glucan synthesis is compromised. In *C. glabrata*, downregulation of Kre5 expression triggers an ER stress response, which subsequently activates the CWI pathway and promotes chitin accumulation [52]. In *C. neoformans*, deletion of Kre5 or Kre6/Skn1 not only alters chitin levels but also disrupts the spatial distribution of its deacetylated derivative, chitosan, which becomes diminished at the septum and aberrantly accumulates in the lateral wall. This suggests that the absence of β-1,6-glucan interferes with the typical spatial arrangement of cell wall components [53]. In *C. auris*, deletion of Kre6a leads to 1.2-fold upregulation of the expression level of the β-1,3-glucan synthase Fsk1 [63]. In *A. fumigatus*, deletion of Kre6 results in elevated levels of β-1,3/1,4-glucan in the conidial cell wall [49], further illustrating the broad and species-specific nature of this compensatory response.

Most GPI-anchored cell wall proteins are covalently linked to β-1,6-glucan via their GPI remnants. Consequently, defects in β-1,6-glucan synthesis directly impair the proper anchoring of these proteins to the cell wall. In *S. cerevisiae*, deletion of *KRE5* or *KRE6* leads to failure of α-agglutinin attachment to the cell wall [92]. Similarly, in *C. albicans*, concurrent deletion of Kre6 and Skn1 results in a dramatic reduction or complete loss of critical virulence-associated GPI proteins, including the hyphal wall protein Hwp1 and the cell surface adhesins (Als3 and Ssa1) [61,62]. In *C. neoformans*, deletion of Kre5 or Kre6/Skn1 diminishes the cell wall localization of the GPI-anchored virulence factor phospholipase B1 (Plb1), with a corresponding increase in its secretion into the culture medium [53]. Collectively, these findings across multiple fungal species support the essential role of β-1,6-glucan in the proper localization and anchoring of GPI proteins to the fungal cell wall.

Deficiencies in β-1,6-glucan synthesis also impair cell wall ultrastructure across multiple fungal species. In *C. albicans*, strains carrying a single gene deletion of *SKN1*, *KRE62*, or *SKN2* retain a normal bilayered wall architecture, although deletion of *SKN1* or *SKN2* alone resulted in a thicker outer layer [48]. By contrast, strains lacking *KRE6* alone, or lacking both *KRE6* and *SKN1*, displayed a heterogeneous and thickened inner layer. Notably, simultaneous deletion of all four genes of the KRE6 family (*KRE6, SKN1, KRE62,* and *SKN2*) completely abolished the outer fibrillar layer while dramatically thickening the inner layer, demonstrating that β-1,6-glucan is essential for bilayered cell wall organization [48,61,62]. Similarly, deletion of Kre6 and Skn1 in *C. neoformans* leads to observable irregularities in cell wall architecture [53]. In *S. cerevisiae*, deletion of Kre9 impairs polarized cell wall development, as evidenced by the inability of mutant cells to form mating projections in response to α-factor [64]. Furthermore, in *C. glabrata*, Kre5 deletion renders the strain hypersensitive to cell wall-perturbing agents like Calcofluor white (CFW) and Congo red (CR) [52]. Similarly, in *C. albicans*, the Kre6 and all multiple mutants aggregate and are hypersensitive to these agents as well as to nikkomycin Z and tunicamycin; notably, the quadruple mutant lacking all four Kre6 homologs shows no growth in the presence of any of these drugs [48]. In *C. auris*, single deletion of Kre6a decreases echinocandin susceptibility, conversely, the *kre6b*Δ mutant overproduces β-1,6-glucan under caspofungin stress and exhibits a weakened cell wall architecture [63]. These findings indicate that the impairment of β-1,6-glucan synthesis disrupts cell wall ultrastructure and compromises cell wall integrity across multiple fungal species, albeit with species-specific phenotypic manifestations.

### 4.3. Function in Fungal Growth and Development

Beyond its structural roles in cell wall integrity and architecture, β-1,6-glucan is also essential for fungal growth and development. Defects in β-1,6-glucan synthesis-associated proteins result in severe growth retardation and morphological abnormalities. Nearly all mutants with pronounced β-1,6-glucan deficiency, including Δ*kre5*, Δ*big1*, Δ*kre6*/*skn1*, exhibit marked growth retardation. Notably, in *S. cerevisiae* and *C. neoformans*, this growth defect can be substantially mitigated by osmotic support [53], suggesting that the compromised cell wall integrity renders these mutants more susceptible to osmotic stress. Beyond growth impairment, most of these mutants also exhibit characteristic morphological anomalies, including cellular enlargement, multipolar budding, impaired cell division, and cell cluster aggregation. For instance, the Δ*kre5* mutant of *S. cerevisiae* displays a multipolar budding morphology [50], while the Δ*kre6*/*skn1* mutant of *C. albicans* shows severe defects in cell separation during the yeast form [61,62]. Collectively, these data establish β-1,6-glucan as an essential determinant of cell wall integrity, osmotic tolerance, normal cell cycle progression and morphogenesis.

Beyond the effects on vegetative growth and cell morphology, β-1,6-glucan deficiency profoundly impairs fungal development, encompassing hyphal morphogenesis, biofilm formation, and the production of infection-related structures. The rapid cell wall remodeling and expansion required for hyphal tip growth is critically dependent on β-1,6-glucan synthesis. In *C. albicans*, among the single *KRE6*-family mutants, only Δ*skn1* displayed a defect in filamentous growth, whereas all multiple mutants examined, including the double mutant Δ*kre6/skn1* and the quadruple mutant Δ*kre6/skn1/kre62/skn2*, exhibited filamentation defects whose severity increased with the number of deleted *KRE6* homologs [48,61,62]. Similarly, hyphal development is impaired in Δ*kre5* and Δ*big1* mutants [51,56]. Given that hyphal proliferation and intercellular adhesion are prerequisites for biofilm development, it is consistent that biofilm formation in *C. albicans* is markedly reduced upon deletion of both Kre6 and Skn1 [61,62]. In the plant pathogenic fungus *C. graminicola,* diminished expression of Kre5 and Kre6 result in a spectrum of aberrant infection-related phenotypes, including anomalous hypha-in-hypha architecture, diminished sporulation, irregular spore size and morphology, compromised spore stability with increased susceptibility to rupture, enlarged and melanized germ tubes, spontaneous appressoria rupture, markedly reduced adhesion, heightened cell wall elasticity, impaired osmotic regulation, and a notable decrease in turgor pressure [54].

In addition, β-1,6-glucan synthesis is a critical determinant of pathogenicity in diverse fungal pathogens. Deletion of the corresponding proteins consistently diminishes or eliminates pathogenicity by compromising cell wall integrity, disrupting the localization of virulence factors, and causing abnormal developmental morphology. In *C. albicans*, deletion of Kre5, Kre6/Skn1/Kre62/Skn2 or Big1 markedly attenuates virulence in murine models [48,51,56,61,62]. Similarly, ablation of Kre5 or Kre6/Skn1 in *C. neoformans* results in complete loss of pathogenicity in mice [53]. In *C. graminicola*, diminished expression of Kre5 or Kre6 leads to complete loss of infectivity on intact maize leaves and greatly reduced ability to proliferate on wounded leaves, causing only minor necrosis at wound edges [54]. The reduction in pathogenicity can be attributed to at least three interconnected mechanisms. First, compromised cell wall integrity, resulting from β-1,6-glucan deficiency, impairs the ability of mutant strains to withstand oxidative stress and host defense proteins. Second, the abnormal localization and function of GPI-anchored virulence proteins, which depend on β-1,6-glucan for proper cell wall anchoring, diminish the adhesion and invasion capacity of the pathogen. Third, defective strain development adversely affects the formation of infection-related structures, thereby compromising overall pathogenicity. Together, these findings establish β-1,6-glucan synthesis as a central hub linking cell wall integrity, virulence factor localization, and morphogenetic competence in fungal pathogenesis.

### 4.4. Immunomodulatory Functions at the Fungus–Host Interface

Beyond its functions in cell wall architecture and fungal growth and development, β-1,6-glucan also acts as a critical signaling molecule at the fungus–host interface. In human pathogenic fungi, β-1,6-glucan is a potent immunomodulatory molecule that actively participates in host immune recognition and fungal immune evasion. Its chain length undergoes highly dynamic remodeling in response to host microenvironmental stimuli. For *C. albicans*, exposure to lactate at the host infection site reduces its content by 72% and decreases its molecular weight from 58 kDa to 19 kDa, whereas gut short-chain fatty acids increase the molecular weight to 64–68 kDa [48]. This structural plasticity directly impacts fungal survival across diverse host niches by modulating the exposure of immunostimulatory epitopes. Immunologically, β-1,6-glucan activates human peripheral blood mononuclear cells (PBMCs) to secrete proinflammatory cytokines [48,93,94]. Despite being a quantitatively minor cell wall component, it activates neutrophils far more efficiently than β-1,3-glucan, driving phagocytosis, ROS burst, and heat shock protein expression [95]. In *Pneumocystis*, β-1,6-glucan triggers tumor necrosis factor alpha (TNF-α) responses through macrophage plasma membrane microdomain-dependent signaling [96,97]. Notably, β-1,6-glucan recognition operates through a complement-dependent, C-type lectin-like receptor (Dectin 1)-independent pathway [98,99,100], distinguishing it from classical β-1,3-glucan recognition mechanisms and suggesting the existence of a dedicated host receptor system for this polymer. It should be noted that part of this immunomodulatory evidence was obtained with β-1,6-glucan preparations whose availability depends on the integrity of the biosynthetic pathway, derived either from cells or cell wall fractions of strains in which β-1,6-glucan synthesis is inhibited, or from purified polymer [95,98]; such studies should therefore be interpreted with the same methodological caveat as digestion-based experiments. Collectively, these findings establish β-1,6-glucan as a multifunctional molecule with three distinct biological roles. Structurally, it is an indispensable cross-linker whose perturbation, whether mediated endogenously or by exogenous β-1,6-glucanases, severely compromises cell wall integrity and fungal viability. Developmentally, it serves as a critical regulator of fungal growth, morphogenesis, and pathogenesis. Immunologically, it acts as a potent signaling molecule at the fungus–host interface, mediating immune recognition and evasion.

## 5. Conclusions and Perspectives

### 5.1. Perspectives on Biosynthesis and Regulation of β-1,6-Glucan

In this review, we have systematically integrated the current understanding of the structure, biosynthesis, and function of β-1,6-glucan in the fungal cell wall. Structurally, β-1,6-glucan is a quantitatively typically minor but architecturally indispensable polysaccharide that functions as a covalent cross-linker, tethering the external mannoprotein layer to the internal chitin–β-1,3-glucan skeletal network, and thereby constituting a mainstay of cell wall integrity and plasticity. Although its absolute abundance and chain architecture differ markedly among species, its fundamental role as the structural bridge of the bilayer cell wall is invariably conserved. Biosynthetically, unlike chitin and β-1,3-glucan, which are polymerized by single plasma membrane-associated synthases, β-1,6-glucan synthesis depends on a multi-protein, multi-compartment cooperative network spanning the ER (Kre5, Big1, Cwh41/Gls1, Rot2/Gls2, and Cne1), the Golgi apparatus (Kre6/Skn1 family homologs), and the cell surface (Kre9, Knh1, Kre1, and Kre11); in vitro reconstitution has further established that polymerization employs UDP-glucose as the direct sugar donor and is positively regulated by the small GTPase Rho1, while strictly depending on preserved cellular integrity. Functionally, two complementary lines of evidence, enzymatic digestion by β-1,6-glucanases and genetic inactivation of the biosynthetic machinery, jointly demonstrate that β-1,6-glucan is essential for cell wall integrity, proper GPI-anchored protein localization, hyphal growth, morphogenesis, and fungal virulence, while simultaneously acting as a potent immunomodulatory signaling molecule at the fungus–host interface. Taken together, these studies establish β-1,6-glucan as a multifunctional molecule with a triad of structural, developmental, and immunomodulatory roles, underscoring its central importance in fungal cell wall biology and host–pathogen interactions.

Based on the cumulative genetic, biochemical, and cell biological evidence reviewed herein, we propose a multi-compartmental model for β-1,6-glucan biosynthesis governed by a multi-layered regulatory network. In the ER, Kre5, Big1, Cwh41/Gls1, Rot2/Gls2, and Cne1 establish the substrate foundation, among which, Kre5 likely generates a glucosylated N-glycan acceptor for subsequent chain elongation [69,70], Big1 facilitates assembly of the synthetic machinery through a partially independent pathway [55], and the glucosidases together with calnexin ensure correct glycoprotein folding, their modest β-1,6-glucan reduction phenotypes (30–50%) indicating facilitatory rather than catalytic roles [58,59]; thus, the ER serves as a substrate preparation gateway, generating a primed acceptor for transport to the Golgi. In the Golgi, Kre6 and Skn1, type II transmembrane proteins with glycoside hydrolase family 16 (GH16) homology, emerge as the most plausible core catalytic components, supported by four converging lines of evidence. First, a screen for resistance to the β-1,6-glucan synthesis inhibitor D75-4590 identified *KRE6* as the primary target [101,102]; second, Kre6 accumulates at polarized growth sites including bud tips and the septation site, an essential requirement for its function, as truncation of its N-terminal cytoplasmic domain abolishes both polarized accumulation and glucan synthesis [103,104]; third, live-cell imaging during cytokinesis defined Kre6 as the likely β-1,6-glucan synthase [105]; fourth, the loss of β-1,6-glucan in Kre6/Skn1 double mutants across *S. cerevisiae*, *C. albicans*, and *C. neoformans* confirms its non-redundant essentiality [48,53,61]. Based on GH16 homology, we propose that Kre6 functions as a transglycosylase that cleaves an existing glucan donor and transfers the glucosyl moiety to form a new β-1,6-linkage, with Skn1 serving as a compensatory paralog. At the cell surface, Kre9, Knh1, Kre1, and Kre11 mediate terminal assembly and cross-linking to GPI-anchored mannoproteins [46,64,75,76]. Regulation operates at multiple levels. Transcriptionally, Skn1 is repressed by Nrg1 during yeast-form growth but derepressed under hypha-inducing conditions [61], while an auto-regulatory loop between *KRE6a* and *KRE6b* in *C. auris* exemplifies fine-tuned transcriptional safeguards within the *KRE* gene family [63]. Post-transcriptionally, the RNA-binding protein Puf4 coordinately regulates the mRNA stability of *SKN1*, *FKS1*, and chitin synthase genes in *C. neoformans* [106,107]. Post-translationally, Rho1p positively regulates synthase activity in a GTP-dependent manner [77], establishing a shared regulatory hub with β-1,3-glucan synthase (Fks1/Rho1) that coordinates glucan deposition in response to cell cycle cues. When β-1,6-glucan synthesis is impaired, the Ca^2+^-calcineurin-PKC-Mkc1 cascade upregulates chitin synthase Chs3 to drive compensatory chitin accumulation that maintains cell viability [62]; the inviability of Kre6/Skn1 Chs3 triple mutants reveals a synthetic lethal interaction with direct therapeutic potential. Additionally, Kre5 downregulation triggers ER stress that activates the CWI pathway to promote chitin accumulation [52]. Collectively, these interconnected mechanisms, including Rho1 signaling, transcriptional control by Nrg1, Puf4-mediated mRNA regulation, and Ca^2+^-calcineurin-PKC stress responses, constitute a multilayered homeostatic network that ensures precise control of β-1,6-glucan content and coordinates its synthesis with other cell wall components in response to developmental and environmental signals.

### 5.2. Conclusions

In summary, the studies reviewed here establish β-1,6-glucan as an architecturally indispensable and functionally versatile component of the fungal cell wall, whose biosynthesis is governed by a conserved multi-compartmental machinery that offers a promising target for antifungal intervention. Nevertheless, several critical problems remain unresolved. Most fundamentally, the core catalytic β-1,6-glucan synthase has not yet been definitively identified, and the precise contributions of the individual accessory proteins remain incompletely defined. Methodologically, β-1,6-glucan synthase activity strictly depends on preserved cellular ultrastructure and cannot be recapitulated in detergent-solubilized systems; this has so far precluded enzyme purification and structural studies, which constitutes a major bottleneck for mechanistic dissection. Interpretation is further complicated by the compensatory remodeling of chitin and β-1,3-glucan that accompanies β-1,6-glucan loss, which obscures direct phenotype attribution; digestion-based approaches additionally carry confounds arising from secondary destabilization of other wall components. The marked species- and lineage-dependent diversity of β-1,6-glucan content and biosynthetic machinery, together with the limited genetic tractability of many basidiomycetes, hampers generalization across fungi. Immunologically, a dedicated, Dectin-1-independent, complement-dependent host receptor for β-1,6-glucan remains to be identified. Looking forward, cryo-electron microscopy of the native membrane-bound complex, genome editing and single-molecule approaches across diverse species, and systematic genetic screens for the core synthase, combined with integrated structural, biochemical, and immunobiological studies, should resolve these bottlenecks. Such progress will deepen our understanding of cell wall biogenesis and establish β-1,6-glucan as a promising, evolutionarily conserved, broad-spectrum antifungal target.

## Figures and Tables

**Table 1:** Diversity in the quantity and chain length of β-1,6-glucan across various fungi.

Fungi Species	Contents (%)	Average Molecular Weight (kDa)	References
*S. cerevisiae*	15	57	[46]
*M. restricta*	70	80	[47]
*C. albicans*	23	43 (hyphal morphology); 70 (caspofungin treatment)	[48]
*A. fumigatus*	NF	NF	[49]

*S. cerevisiae*, *Saccharomyces cerevisiae; M. restricta*, *Malassezia restricta; C. albicans*, *Candida albicans; A. fumigatus*, *Aspergillus fumigatus*. NF, not found. Values in the content column represent the percentage of β-1,6-glucan content relative to the total dry weight of the cell wall.

**Table 3:** Endogenous and exogenous β-1,6-glucanases reveal the indispensable role of β-1,6-glucan in the fungal cell wall.

Category	Source	Enzyme	Phenotypic Consequences	Biological Significance	References
Endogenous	*L.* *edodes*	LePus30A	Expression markedly elevated during postharvest autolysis	β-1,6-glucan degradation is required for fruiting body senescence and autolysis	[79,80]
	*C.* *cinerea*	GH30A	Endogenous hydrolysis of β-1,6-glucan in the stipe cell wall restores cell wall extensibility of heat-inactivated stipes	β-1,6-Glucan-chitin cross-links are essential for stipe elongation	[81,82]
	*M.* *oryzae*	MoGlu16	Its deletion reduced mycelial growth, altered cell wall structure, attenuated pathogenicity	β-1,6-glucan is required for growth, cell wall integrity and pathogenicity	[83,84]
	*V. fungicola*	VfGlu1	Its deletion markedly diminishes pathogenicity toward *Agaricus bisporus*	β-1,6-glucan turnover is crucial for infection fitness	[8,85]
Exogenous	*Corallococcus* sp. EGB	GluM	Its treatment against *M. oryzae* resulted in aberrant hyphae, altered chitin distribution, enhanced membrane permeability and growth suppression	β-1,6-glucan is indispensable for cell wall integrity and viability	[86,87]
	*Flavobacterium* sp. NAU1659	FlGlu30	Its treatment compromised the cell wall of *M. oryzae*, induced membrane damage, ROS accumulation, and inhibited conidial germination and appressorium formation	β-1,6-glucan is essential for conidial germination and appressorium development	[88]
	*T. harzianum*	BGN16.3/Neg1	They efficiently suppress fungal growth via synergism with chitinases	β-1,6-glucan is a key cross-linker; its removal destabilizes the cell wall	[89,90,91]

*L. edodes*, *Lentinula edodes*; *C. cinerea*, *Coprinopsis cinerea*; *M. oryzae*, *Magnaporthe oryzae*; *V. fungicola*, *Verticillium fungicola*; *T. harzianum*, *Trichoderma harzianum*.

## Data Availability

No new data were created or analyzed in this study. Data sharing is not applicable.

## References

[1] Gow N.A., Lenardon M.D. (2023). Architecture of the dynamic fungal cell wall. Nat. Rev. Microbiol..

[2] Gautam I., Ankur A., Singh K., Jacob A., Doering T.L., Gow N.A.R., Latgé J.P., Wang T. (2025). Breaking down the wall: Solid-state NMR illuminates how fungi build and remodel diverse cell walls. PLoS Pathog..

[3] Gow N.A. (2025). Fungal cell wall biogenesis: Structural complexity, regulation and inhibition. Fungal Genet. Biol..

[4] Fernando L.D., Pérez-Llano Y., Dickwella Widanage M.C., Jacob A., Martínez-Ávila L., Lipton A.S., Gunde-Cimerman N., Latgé J.P., Batista-García R.A., Wang T. (2023). Structural adaptation of fungal cell wall in hypersaline environment. Nat. Commun..

[5] Shree A., Pal S., Verma P.K. (2024). Structural diversification of fungal cell wall in response to the stress signaling and remodeling during fungal pathogenesis. Physiol. Mol. Biol. Plants.

[6] Zhao M., Huang H., Li B., Pan Y., Wang C., Du W., Wang W., Wang Y., Mao X., Kong X. (2025). Preparation of *Auricularia auricula*-derived immune modulators and alleviation of cyclophosphamide-induced immune suppression and intestinal microbiota dysbiosis in mice. Life.

[7] Li Y., Gong K., Wang X., Sun Z., Ding F. (2025). Heat shock transcription factors as central integrators of plant stress responses: From thermotolerance to multi-stress resilience. Biology.

[8] Gea F.J., Navarro M.J., Santos M., Diánez F., Carrasco J. (2021). Control of fungal diseases in mushroom crops while dealing with fungicide resistance: A review. Microorganisms.

[9] Yugueros S.I., Peláez J., Stajich J.E., Fuertes-Rabanal M., Sánchez-Vallet A., Largo-Gosens A., Mélida H. (2024). Study of fungal cell wall evolution through its monosaccharide composition: An insight into fungal species interacting with plants. Cell Surf..

[10] Latgé J.P., Wang T. (2022). Modern biophysics redefines our understanding of fungal cell wall structure, complexity, and dynamics. mBio.

[11] Gow N.A.R., Latgé J.-P., Munro C.A. (2017). The fungal cell wall: Structure, biosynthesis, and function. Microbiol. Spectr..

[12] Yu Y., Wen Q., Song A., Liu Y., Wang F., Jiang B. (2022). Isolation and immune activity of a new acidic *Cordyceps militaris* exopolysaccharide. Int. J. Biol. Macromol..

[13] Zhang Y., Ding Z., Chen X., Wen M., Wang Q., Wang Z. (2023). Effects of oligosaccharide fermentation on canine gut microbiota and fermentation metabolites in an in vitro fecal fermentation model. Fermentation.

[14] Fesel P.H., Zuccaro A. (2016). β-glucan: Crucial component of the fungal cell wall and elusive MAMP in plants. Fungal Genet. Biol..

[15] Wang Y., Xie T., Ma C., Zhao Y., Li J., Li Z., Ye X. (2024). Biochemical characterization and antifungal activity of a recombinant β-1,3-glucanase FlGluA from *Flavobacterium* sp. NAU1659. Protein Expr. Purif..

[16] Liu J.J., Hou Y.K., Wang X., Zhou X.T., Yin J.Y., Nie S.P. (2024). Recent advances in the biosynthesis of fungal glucan structural diversity. Carbohydr. Polym..

[17] Ye X., Xu C., Xie T., Zhang Y., Zhao Y., Xia C., Li Z., Huang Y., Fan J., Cao H. (2023). Myxobacterial outer membrane β-1,6-glucanase induced the cell death of *Fusarium oxysporum* by destroying the cell wall integrity. Appl. Environ. Microbiol..

[18] Kang X., Kirui A., Muszyński A., Widanage M.C.D., Chen A., Azadi P., Wang P., Mentink-Vigier F., Wang T. (2018). Molecular architecture of fungal cell walls revealed by solid-state NMR. Nat. Commun..

[19] Brain L., Bleackley M., Doblin M.S., Anderson M. (2025). Fungal chitin synthases: Structure, function, and regulation. J. Fungi.

[20] Xu L., Wen T.-T., Chen C.-Y., Sun L., Liu Z.-Y., Wang H.-Y., Sun W.-J., Cui F.-J. (2026). Chitin synthases in fungi: A comprehensive review of classification, catalytic mechanism, and functional roles. J. Agric. Food Chem..

[21] Zhou X., Huang Y., Liu Y., Pan D., Zhang Y. (2024). Continuous production of chitin oligosaccharides utilizing an optimized enzyme production-adsorption-enzymolysis-product separation (EAES) system. Fermentation.

[22] Obara K., Okada H., Tan G., Ohnuki S., Suzuki G., Ohtake H., Kubo K., Ghanegolmohammadi F., Ishizaka S., Yashiroda Y. (2026). Role of Pbr1, a putative oxidoreductase, in the ER quality control and folding of yeast Fks1 glucan synthase. Proc. Natl. Acad. Sci. USA.

[23] Liu W., Sekiguchi H., Li S.C., Isuhuaylas L.A.V., Yamanaka D., Itto-Nakama K., Noda Y., Boone C., Yashiroda Y., Ohya Y. (2026). The novel antifungal agent NPD2560 perturbs the Rho1-centered signaling network to induce a cell wall integrity response. Microbiol. Spectr..

[24] Li J., Liu H., Li J., Liu J., Dai X., Zhu A., Xiao Q., Qian W., Li H., Guo L. (2025). Cryo-EM structure of the β-1,3-glucan synthase FKS1-Rho1 complex. Nat. Commun..

[25] Yoshimi A., Miyazawa K., Abe K. (2017). Function and biosynthesis of cell wall α-1,3-glucan in fungi. J. Fungi.

[26] Ost K.J., Student M., Cord-Landwehr S., Moerschbacher B.M., Ram A.F., Dirks-Hofmeister M.E. (2025). Cell walls of filamentous fungi–challenges and opportunities for biotechnology. Appl. Microbiol. Biotechnol..

[27] Miyazawa K., Yoshimi A., Kasahara S., Sugahara A., Koizumi A., Yano S., Kimura S., Iwata T., Sano M., Abe K. (2018). Molecular mass and localization of α-1,3-glucan in cell wall control the degree of hyphal aggregation in liquid culture of *Aspergillus nidulans*. Front. Microbiol..

[28] Baek K.-R., Ramakrishnan S.R., Kim S.-J., Seo S.-O. (2024). Yeast cell wall mannan structural features, biological activities, and production strategies. Heliyon.

[29] Chraniuk P., Bzducha-Wróbel A. (2025). Functional properties of yeast mannoproteins—Current knowledge and future perspectives. Fermentation.

[30] Loza L., Doering T.L. (2024). A fungal protein organizes both glycogen and cell wall glucans. Proc. Natl. Acad. Sci. USA.

[31] Dong Y., Liu X., An Y., Liu M., Han J., Sun B. (2020). Potent arylamide derivatives as dual-target antifungal agents: Design, synthesis, biological evaluation, and molecular docking studies. Bioorg. Chem..

[32] Hoenigl M., Arastehfar A., Arendrup M.C., Brüggemann R., Carvalho A., Chiller T., Chen S., Egger M., Feys S., Gangneux J.P. (2024). Novel antifungals and treatment approaches to tackle resistance and improve outcomes of invasive fungal disease. Clin. Microbiol. Rev..

[33] Hu X., Yang P., Chai C., Liu J., Sun H., Wu Y., Zhang M., Zhang M., Liu X., Yu H. (2023). Structural and mechanistic insights into fungal β-1,3-glucan synthase FKS1. Nature.

[34] Wei Y., Wang J., Tang N., Lin Z., Li W., Xu Y., Guo L. (2025). Caspofungin paradoxical growth in *Candida albicans* requires stress pathway activation and promotes unstable echinocandin resistance mediated by aneuploidy. Front. Cell. Infect. Microbiol..

[35] Lee Y., Robbins N., Cowen L.E. (2023). Molecular mechanisms governing antifungal drug resistance. npj Antimicrob. Resist..

[36] Puumala E., Fallah S., Robbins N., Cowen L.E. (2024). Advancements and challenges in antifungal therapeutic development. Clin. Microbiol. Rev..

[37] Maves R.C., Fowler M.N. (2025). New and emerging drugs for the treatment of invasive fungal infections. Expert Opin. Pharmacother..

[38] Hetta H.F., Melhem T., Aljohani H.M., Salama A., Ahmed R., Elfadil H., Alanazi F.E., Ramadan Y.N., Battah B., Rottura M. (2025). Beyond conventional antifungals: Combating resistance through novel therapeutic pathways. Pharmaceuticals.

[39] Ruiz-Herrera J., Ortiz-Castellanos L. (2019). Cell wall glucans of fungi. A review. Cell Surf..

[40] Plaza V., Silva-Moreno E., Castillo L. (2020). Breakpoint: Cell wall and glycoproteins and their crucial role in the phytopathogenic fungi infection. Curr. Protein Pept. Sci..

[41] Kapteyn J.C., Montijin R.C., Vink E., de la Cruz J., Llobell A., Douwes J.E., Shimoi H., Lipke P.N., Klis F.M. (1996). Retention of *Saccharomyces cerevisiae* cell wall proteins through a phosphodiester-linked β-1,3-/β-l,6-glucan heteropolymer. Glycobiology.

[42] Soler A., de la Cruz J., Llobell A. (1999). Detection of β-1,6-glucanase isozymes from *Trichoderma* strains in sodium dodecyl sulphate–polyacrylamide gel electrophoresis and isoelectrofocusing gels. J. Microbiol. Methods.

[43] Ibe C., Munro C.A. (2021). Fungal cell wall: An underexploited target for antifungal therapies. PLoS Pathog..

[44] Brown A.J. (2023). Fungal resilience and host–pathogen interactions: Future perspectives and opportunities. Parasite Immunol..

[45] Lima S.L., Colombo A.L., de Almeida Junior J.N. (2019). Fungal cell wall: Emerging antifungals and drug resistance. Front. Microbiol..

[46] Shahinian S., Bussey H. (2000). β-1,6-Glucan synthesis in *Saccharomyces cerevisiae*. Mol. Microbiol..

[47] Stalhberger T., Simenel C., Clavaud C., Eijsink V.G., Jourdain R., Delepierre M., Latgé J.-P., Breton L., Fontaine T. (2014). Chemical organization of the cell wall polysaccharide core of *Malassezia restricta*. J. Biol. Chem..

[48] Bekirian C., Valsecchi I., Bachellier-Bassi S., Scandola C., Guijarro J.I., Chauvel M., Mourer T., Gow N.A., Aimanianda V.K., D’Enfert C. (2024). β-1,6-glucan plays a central role in the structure and remodeling of the bilaminate fungal cell wall. eLife.

[49] Singh K., Henry C., Mouyna I., Beauvais A., Xu Y., Karai A., van Rhijn N., Latgé J.-P., Wang T. (2025). Kre6-dependent β-1,6-glucan biosynthesis only occurs in the conidium of *Aspergillus fumigatus*. mSphere.

[50] Meaden P., Hill K., Wagner J., Slipetz D., Sommer S., Bussey H. (1990). The yeast *KRE5* gene encodes a probable endoplasmic reticulum protein required for (1-6)-beta-D-glucan synthesis and normal cell growth. Mol. Cell. Biol..

[51] Herrero A.B., Magnelli P., Mansour M.K., Levitz S.M., Bussey H., Abeijon C. (2004). *KRE5* gene null mutant strains of *Candida albicans* are avirulent and have altered cell wall composition and hypha formation properties. Eukaryot. Cell.

[52] Tanaka Y., Sasaki M., Ito F., Aoyama T., Sato-Okamoto M., Takahashi-Nakaguchi A., Chibana H., Shibata N. (2016). *KRE5* suppression induces cell wall stress and alternative ER stress response required for maintaining cell wall integrity in *Candida glabrata*. PLoS ONE.

[53] Gilbert N.M., Donlin M.J., Gerik K.J., Specht C.A., Djordjevic J.T., Wilson C.F., Sorrell T.C., Lodge J.K. (2010). *KRE* genes are required for β-1,6-glucan synthesis, maintenance of capsule architecture and cell wall protein anchoring in *Cryptococcus neoformans*. Mol. Microbiol..

[54] Oliveira-Garcia E., Deising H.B. (2016). Attenuation of PAMP-triggered immunity in maize requires down-regulation of the key β-1,6-glucan synthesis genes *KRE5* and *KRE6* in biotrophic hyphae of *Colletotrichum graminicola*. Plant J..

[55] Azuma M., Levinson J.N., Pagé N., Bussey H. (2002). *Saccharomyces cerevisiae* Big1p, a putative endoplasmic reticulum membrane protein required for normal levels of cell wall β-1,6-glucan. Yeast.

[56] Umeyama T., Kaneko A., Watanabe H., Hirai A., Uehara Y., Niimi M., Azuma M. (2006). Deletion of the *CaBIG1* gene reduces β-1,6-glucan synthesis, filamentation, adhesion, and virulence in *Candida albicans*. Infect. Immun..

[57] Jiang B., Sheraton J., Ram A., Dijkgraaf G., Klis F.M., Bussey H. (1996). *CWH41* encodes a novel endoplasmic reticulum membrane N-glycoprotein involved in beta 1,6-glucan assembly. J. Bacteriol..

[58] Trombetta E.S., Simons J.F., Helenius A. (1996). Endoplasmic reticulum glucosidase II is composed of a catalytic subunit, conserved from yeast to mammals, and a tightly bound noncatalytic HDEL-containing subunit. J. Biol. Chem..

[59] Tanaka Y., Sasaki M., Ito F., Aoyama T., Sato-Okamoto M., Takahashi-Nakaguchi A., Chibana H., Shibata N. (2018). Cooperation between ER stress and calcineurin signaling contributes to the maintenance of cell wall integrity in *Candida glabrata*. Fungal Biol..

[60] Roemer T., Paravicini G., Payton M.A., Bussey H. (1994). Characterization of the yeast (1-6)-beta-glucan biosynthetic components, Kre6p and Skn1p, and genetic interactions between the PKC1 pathway and extracellular matrix assembly. J. Cell Biol..

[61] Han Q., Wang N., Yao G., Mu C., Wang Y., Sang J. (2019). Blocking β-1,6-glucan synthesis by deleting *KRE6* and *SKN1* attenuates the virulence of *Candida albicans*. Mol. Microbiol..

[62] Han Q., Wang N., Pan C., Wang Y., Sang J. (2019). Elevation of cell wall chitin via Ca^2+^–calcineurin-mediated PKC signaling pathway maintains the viability of *Candida albicans* in the absence of β-1,6-glucan synthesis. Mol. Microbiol..

[63] Dickwella Widanage M.C., Singh K., Li J., Yarava J.R., Scott F.J., Xu Y., Gow N.A.R., Mentink-Vigier F., Wang P., Lamoth F. (2025). Distinct echinocandin responses of *Candida albicans* and *Candida auris* cell walls revealed by solid-state NMR. Nat. Commun..

[64] Brown J.L., Bussey H. (1993). The yeast *KRE9* gene encodes an O glycoprotein involved in cell surface beta-glucan assembly. Mol. Cell. Biol..

[65] Boone C., Sommer S.S., Hensel A., Bussey H. (1990). Yeast *KRE* genes provide evidence for a pathway of cell wall beta-glucan assembly. J. Cell Biol..

[66] Dijkgraaf G.J., Brown J.L., Bussey H. (1996). The *KNH1* gene of *Saccharomyces cerevisiae* is a functional homolog of *KRE9*. Yeast.

[67] Tax G., Guay K.P., Pantalone L., Ceci M., Soldà T., Hitchman C.J., Hill J.C., Vasiljević S., Lia A., Modenutti C.P. (2024). Rescue of secretion of rare-disease-associated misfolded mutant glycoproteins in UGGT1 knock-out mammalian cells. Traffic.

[68] Tessier D.C., Dignard D., Zapun A., Radominska-Pandya A., Parodi A.J., Bergeron J.J., Thomas D.Y. (2000). Cloning and characterization of mammalian UDP-glucose glycoprotein: Glucosyltransferase and the development of a specific substrate for this enzyme. Glycobiology.

[69] Wang N., Seko A., Takeda Y., Ito Y. (2020). Glycan dependent refolding activity of ER glucosyltransferase (UGGT). Biochim. Biophys. Acta Gen. Subj..

[70] Shahinian S., Dijkgraaf G.J., Sdicu A.-M., Thomas D.Y., Jakob C.A., Aebi M., Bussey H. (1998). Involvement of protein N-glycosyl chain glucosylation and processing in the biosynthesis of cell wall β-1,6-glucan of *Saccharomyces cerevisiae*. Genetics.

[71] Sharninghausen R., Hwang J., Dennison D.D., Baldridge R.D. (2024). Identification of ERAD-dependent degrons for the endoplasmic reticulum lumen. eLife.

[72] Parlati F., Dominguez M., Bergeron J.J., Thomas D.Y. (1995). *Saccharomyces cerevisiae CNE1* encodes an endoplasmic reticulum (ER) membrane protein with sequence similarity to calnexin and calreticulin and functions as a constituent of the er quality control apparatus. J. Biol. Chem..

[73] Malavia-Jones D., Farrer R.A., Stappers M.H.T., Edmondson M.B., Borman A.M., Johnson E.M., Lipke P.N., Gow N.A.R. (2023). Strain and temperature dependent aggregation of *Candida auris* is attenuated by inhibition of surface amyloid proteins. Cell Surf..

[74] Roemer T., Bussey H. (1995). Yeast Kre1p is a cell surface O-glycoprotein. Mol. Gen. Genet..

[75] Sacher M., Jiang Y., Barrowman J., Scarpa A., Burston J., Zhang L., Schieltz D., Yates J.R., Abeliovich H., Ferro-Novick S. (1998). TRAPP, a highly conserved novel complex on the cis-Golgi that mediates vesicle docking and fusion. EMBO J..

[76] Brown J.L., Kossaczka Z., Jiang B., Bussey H. (1993). A mutational analysis of killer toxin resistance in *Saccharomyces cerevisiae* identifies new genes involved in cell wall (1-6)-beta-glucan synthesis. Genetics.

[77] Vink E., Rodriguez-Suarez R.J., Gérard-Vincent M., Ribas J.C., de Nobel H., van den Ende H., Durán A., Klis F.M., Bussey H. (2004). An in vitro assay for (1-6)-β-D-glucan synthesis in *Saccharomyces cerevisiae*. Yeast.

[78] Aimanianda V., Clavaud C., Simenel C., Fontaine T., Delepierre M., Latge J.-P. (2009). Cell wall β-(1,6)-glucan of *Saccharomyces cerevisiae*: Structural characterization and in situ synthesis. J. Biol. Chem..

[79] Konno N., Sakamoto Y. (2011). An endo-β-1,6-glucanase involved in *Lentinula edodes* fruiting body autolysis. Appl. Microbiol. Biotechnol..

[80] Kobayashi N., Wada N., Yokoyama H., Tanaka Y., Suzuki T., Habu N., Konno N. (2023). Extracellular enzymes secreted in the mycelial block of *Lentinula edodes* during hyphal growth. AMB Express.

[81] Liu X., Wang R., Bi J., Kang L., Zhou J., Duan B., Liu Z., Yuan S. (2020). A novel endo-β-1,6-glucanase from the mushroom *Coprinopsis cinerea* and its application in studying of cross-linking of β-1,6-glucan and the wall extensibility in stipe cell walls. Int. J. Biol. Macromol..

[82] Duan B., Bi J., Liu Z., Zhu Y., Zhang Z., Sun L., Kang L., Wu B., Zhang W., Yuan S. (2025). Forms of chitin-polysaccharide cross-linking in the *Coprinopsis cinerea* stipe cell wall. Carbohydr. Polym..

[83] Wang Y., Li J., Zhang Y., Chen K., Zhao Y., Zhao Z., Li Z. (2026). The β-1,6-glucanase MoGlu16 is required for mycelia growth and pathogenicity of *Magnaporthe oryzae*. Curr. Microbiol..

[84] Wang Y., Li D., Li Z., Cui Z., Ye X. (2024). Functional analysis of a novel endo-β-1,6-glucanase MoGlu16 and its application in detecting cell wall β-1,6-glucan of *Magnaporthe oryzae*. Front. Microbiol..

[85] Amey R.C., Mills P.R., Bailey A., Foster G.D. (2003). Investigating the role of a *Verticillium fungicola* β-1,6-glucanase during infection of *Agaricus bisporus* using targeted gene disruption. Fungal Genet. Biol..

[86] Li Z., Wang J., Lin K., Liu M., Wang J., Zhang L., Xia C., Liu L., Zhang B., Yangzong Y. (2023). Insights into the antifungal properties of myxobacteria outer membrane β-1,6-glucanase. J. Agric. Food Chem..

[87] Li Z., Ye X., Liu M., Xia C., Zhang L., Luo X., Wang T., Chen Y., Zhao Y., Qiao Y. (2019). A novel outer membrane β-1,6-glucanase is deployed in the predation of fungi by myxobacteria. ISME J..

[88] Xie T., Shen J., Geng Z., Wu F., Dong Y., Cui Z., Liang Y., Ye X. (2024). Antifungal characterizations of a novel endo-β-1,6-glucanase from *Flavobacterium* sp. NAU1659. Appl. Microbiol. Biotechnol..

[89] Montero M., Sanz L., Rey M., Monte E., Llobell A. (2005). BGN16.3, a novel acidic β-1,6-glucanase from mycoparasitic fungus *Trichoderma harzianum* CECT 2413. FEBS J..

[90] Liu J., Wang Y., Lu S., Liu X., Zhang H., Wang W. (2024). Neg1 overexpression in *Trichoderma harzianum* T4 enhanced its ability to produce spores and antagonistic activity against phytopathogenic fungi. Arch. Microbiol..

[91] Tyśkiewicz R., Nowak A., Ozimek E., Jaroszuk-Ściseł J. (2022). Trichoderma: The current status of its application in agriculture for the biocontrol of fungal phytopathogens and stimulation of plant growth. Int. J. Mol. Sci..

[92] Lu C.-F., Montijn R.C., Brown J.L., Klis F., Kurjan J., Bussey H., Lipke P.N. (1995). Glycosyl phosphatidylinositol-dependent cross-linking of alpha-agglutinin and beta 1,6-glucan in the *Saccharomyces cerevisiae* cell wall. J. Cell Biol..

[93] Vuscan P., Röring R.J., Kischkel B., Tintoré M., Cuñé J., de Lecea C., Joosten L.A.B., Netea M.G. (2025). Effect of *Saccharomyces cerevisiae* β-glucan on the T helper cytokine profile. Cytokine.

[94] De Marco Castro E., Calder P.C., Roche H.M. (2021). β-1,3/1,6-Glucans and immunity: State of the art and future directions. Mol. Nutr. Food Res..

[95] Rubin-Bejerano I., Abeijon C., Magnelli P., Grisafi P., Fink G.R. (2007). Phagocytosis by human neutrophils is stimulated by a unique fungal cell wall component. Cell Host Microbe.

[96] Kottom T.J., Hebrink D.M., Jenson P.E., Gudmundsson G., Limper A.H. (2015). Evidence for proinflammatory β-1,6 glucans in the *Pneumocystis carinii* cell wall. Infect. Immun..

[97] Wang M., Zhang Z., Dong X., Zhu B. (2023). Targeting β-glucans, vital components of the *Pneumocystis* cell wall. Front. Immunol..

[98] Singh R.P., Bhardwaj A. (2023). β-glucans: A potential source for maintaining gut microbiota and the immune system. Front. Nutr..

[99] Mata-Martínez P., Bergón-Gutiérrez M., Del Fresno C. (2022). Dectin-1 signaling update: New perspectives for trained immunity. Front. Immunol..

[100] Noorbakhsh Varnosfaderani S.M., Ebrahimzadeh F., Akbari Oryani M., Khalili S., Almasi F., Mosaddeghi Heris R., Payandeh Z., Li C., Nabi Afjadi M., Alagheband Bahrami A. (2024). Potential promising anticancer applications of β-glucans: A review. Biosci. Rep..

[101] Kitamura A., Someya K., Hata M., Nakajima R., Takemura M. (2009). Discovery of a small-molecule inhibitor of β-1,6-glucan synthesis. Antimicrob. Agents Chemother..

[102] Kubo K., Itto-Nakama K., Ohnuki S., Yashiroda Y., Li S.C., Kimura H., Kawamura Y., Shimamoto Y., Tominaga K.I., Yamanaka D. (2022). Jerveratrum-type steroidal alkaloids inhibit β-1,6-glucan biosynthesis in fungal cell walls. Microbiol. Spectr..

[103] Kurita T., Noda Y., Takagi T., Osumi M., Yoda K. (2011). Kre6 protein essential for yeast cell wall β-1,6-glucan synthesis accumulates at sites of polarized growth. J. Biol. Chem..

[104] Kshirsagar R., Munhoven A., Tran Nguyen T.M., Ehrenhofer-Murray A.E. (2024). A role for β-1,6- and β-1,3-glucans in kinetochore function in *Saccharomyces cerevisiae*. Genetics.

[105] Okada H., MacTaggart B., Ohya Y., Bi E. (2021). The kinetic landscape and interplay of protein networks in cytokinesis. iScience.

[106] Kalem M.C., Subbiah H., Leipheimer J., Glazier V.E., Panepinto J.C. (2021). Puf4 mediates post-transcriptional regulation of cell wall biosynthesis and caspofungin resistance in *Cryptococcus neoformans*. mBio.

[107] Gonzalez-Jimenez I., Keniya M.V., Aptekmann A.A., Quinteros C., Wilkerson A., Arastehfar A., Daneshnia F., Perlin D.S., Shor E. (2025). Expression of 1,3-β-glucan synthase subunits in *Candida glabrata* is regulated by the cell cycle and growth conditions and at both transcriptional and post-transcriptional levels. Antimicrob. Agents Chemother..

